# Minor Ginsenoside Rg2 and Rh1 Attenuates LPS-Induced Acute Liver and Kidney Damages via Downregulating Activation of TLR4-STAT1 and Inflammatory Cytokine Production in Macrophages

**DOI:** 10.3390/ijms21186656

**Published:** 2020-09-11

**Authors:** Diem Thi Ngoc Huynh, Naehwan Baek, Sohyun Sim, Chang-Seon Myung, Kyung-Sun Heo

**Affiliations:** 1 College of Pharmacy and Institute of Drug Research and Development, Chungnam National University, Daejeon 34134, Korea; ngocdiemphar@gmail.com (D.T.N.H.); ghks5139@cnu.ac.kr (N.B.); sohyun.sim@keco.or.kr (S.S.); cm8r@cnu.ac.kr (C.-S.M.); 2 Department of Chemicals Assessment, Korea Environment Corporation, Incheon 404-708, Korea

**Keywords:** LPS, ginsenoside Rg2 and Rh1, NO, NF-κB, STAT1, LPS-induced mice

## Abstract

Ginsenosides have been reported to have various biological effects, such as immune regulation and anticancer activity. In this study, we investigated the anti-inflammatory role of a combination of Rg2 and Rh1, which are minor ginsenosides, in lipopolysaccharide (LPS)-stimulated inflammation. In vitro experiments were performed using the RAW264.7 cell line, and an in vivo model of inflammation was established using LPS-treated ICR mice. We employed Griess assay, 3-(4,5-dimethylthiazol-2-yl)-2,5-diphenyltetrazolium bromide (MTT) assay, quantitative reverse transcriptase-polymerase chain reaction, western blotting, immunofluorescence staining, and hematoxylin and eosin staining to evaluate the effect of Rg2 and Rh1. We found that Rg2 and Rh1 significantly decreased LPS-induced major inflammatory mediator production, inducible-nitric oxide synthase expression, and nitric oxide production in macrophages. Moreover, Rg2 and Rh1 combination treatment inhibited the binding of LPS to toll-like receptor 4 (TLR4) on peritoneal macrophages. Therefore, the combination of ginsenoside Rg2 and Rh1 suppressed inflammation by abolishing the binding of LPS to TLR4, thereby inhibiting the TLR4-mediated signaling pathway. The combined ginsenoside synergistically blocked LPS-mediated PKCδ translocation to the plasma membrane, resulting in p38-STAT1 activation and NF-κB translocation. In addition, mRNA levels of pro-inflammatory cytokines, including TNF-α, IL-1β, and IFN-β, were significantly decreased by combined ginsenoside treatment. Notably, the 20 mg/kg ginsenoside treatment significantly reduced LPS-induced acute tissue inflammation levels in vivo, as indicated by the tissue histological damage scores and the levels of biochemical markers for liver and kidney function from mouse serum. These results suggest that the minor ginsenosides Rg2 and Rh1 may play a key role in prevention of LPS-induced acute inflammation and tissue damage.

## 1. Introduction

Sepsis, which is a systemic inflammatory response to an infection, leads to organ dysfunction and death [1]. During septic shock, the inflammatory response causes hepatocyte dysfunction. Additionally, the increase in pro-inflammatory cytokines such as TNF-α, IL-1β, and IL-6 inhibits thrombosis regulation [2]. Acute liver injury and kidney damage are often associated with sepsis, which can be life-threatening [3]. Moreover, sepsis often affects the kidneys, resulting in sepsis-associated acute kidney injury that increases the risk of in-hospital death [4].

Lipopolysaccharide (LPS), a major component of the outer membrane of gram-negative bacteria, is one of the factors that trigger systemic inflammatory response [5]. Macrophages, a main type of antigen presenting cell, are widely distributed in the body and play a critical role in modulating the inflammatory response using various receptors [6]. Toll-like receptor (TLR) 4 is one of the receptors that is activated by LPS [7]. The binding between LPS and TLR4 leads to the activation of the mitogen-activated protein kinase (MAPK) pathway, which includes extracellular signal-regulated kinase (ERK), p38, and c-Jun NH_2_-terminal kinase (JNK) [8].

In addition to kinase activation, nuclear factor kappa B (NF-κB), which is a transcription factor composed of p50 and p65 subunits, is involved in the inflammatory signaling pathway [9]. NF-κB transactivation is crucial for the upregulation of various pro-inflammatory cytokines, including tumor necrosis factor-alpha (TNF-α) and interleukin-1 beta (IL-1β) [10]. It has been reported that the Janus kinases-signal transducer and activator of transcription (JAK-STAT) protein signaling pathway is associated with increased pro-inflammatory mediators, such as inducible-nitric oxide synthase (iNOS), that produce nitric oxide (NO) [11].

Ginsenosides, the main active components of *Panax ginseng* Mayer (*P. ginseng*), are mainly classified into protopanaxadiol (PPD; Rb1, Rb2, Rb3, Rc, Rd, Rg3, and Rh2)- and protopanaxatriol (PPT; Re, Rg1, Rg2, and Rh1)-type saponins [12]. They have been shown to have various pharmacological effects, including anticancer and anti-inflammatory effects, and beneficial effects against cardiovascular diseases and immunodeficiency [13,14,15,16]. Among them, Rg1 attenuates hepatocellular apoptosis and inflammatory response in ischemia–reperfusion injury mouse models [17]. Ginsenoside Rb1 inhibits acute intestinal ischemia reperfusion-induced renal injury by activating the nuclear erythroid 2-related factor 2/antioxidant response element pathway [18]. Another ginsenoside, Rk3, has been revealed to exert the inhibitory effect on chronic alcohol-induced liver injury in mice by suppressing inflammation, oxidative stress, and apoptosis [19]. Additionally, 20(S)-ginsenoside Rg3 prevents hepatic and renal injury induced by LPS and D-Galactose through the regulation of oxidative stress-mediated apoptosis [20,21]. Rg3 also exerts an inhibitory effect on hepatic fibrosis via the reduction of inflammation-mediated autophagy [22]. Particularly, PPT-type ginseng saponins Rg2 and Rh1 are classified as minor ginsenosides, which are produced by hydrolysis of the sugar moieties of major ginsenosides [23]. In previous studies, Rg2 has been shown to exert antidepressant-like and anti-adipogenesis effects [24,25], and Rh1 has been shown to exert anti-inflammatory effects [26]. Notably, Rg2 and Rh1 are associated with various signaling pathways such as AMP-activated protein kinase (AMPK), MAPK, protein kinase B (Akt), as well as JAK/STAT in different cell types [25,27,28,29,30]. In particular, ginsenoside Rg2 inhibits adipogenesis in 3T3-L1 preadipocytes and suppresses obesity in obese mice induced by high-fat-diet through the AMPK pathway [25]. Moreover, Rg2 reduces hepatic glucose production in HepG2 cells by inactivating glycogen synthase kinase 3 beta via AMPK pathway [29]. Additionally, ginsenoside Rh1 has the ability to abolish HIV-1 infected macrophages via the inhibition of the PDK1/Akt pathway [28]. Rh1 also attenuates iNOS expression by inhibiting IFN-γ-induced JAK/STAT and ERK signaling pathways in IFN-γ-stimulated BV2 microglial cells [30]. Rh1 also effectively suppresses the invasion and the migration of glioma cells through blocking MAPK and PI3K/Akt signaling pathways and downstream transcription factors including NF-κB and AP-1 [27]. Minor ginsenoside Rg2 and Rh1 are known to be converted from the major ginsenoside Re and Rg1 [31]. However, the anti-inflammatory effects of the combination of minor ginsenoside Rg2 and Rh1 and their underlying signaling pathway have not been thoroughly studied. Additionally, MAPK and JAK/STAT pathways, which are highly associated with inflammation, are the main targets to examine the mechanisms related to the effects of Rg2 and Rh1 combination. 

In this study, we investigated the preventative effects of Rg2 and Rh1 on inflammatory diseases and provided insight on their molecular mechanisms in vitro and in vivo. Rg2 and Rh1 inhibited p38 MAPK and JAK-STAT1 activation and NF-κB translocation to the nucleus in LPS-stimulated macrophages. In concordance with in vitro experimental findings, Rg2 and Rh1 reduced LPS-induced acute liver and kidney damage by downregulation of cytokine production.

## 2. Results

### 2.1. Effect of the Ginsenosides Rg2 and Rh1 on Cell Viability and Inflammatory Mediator Production

**Figure 1:**
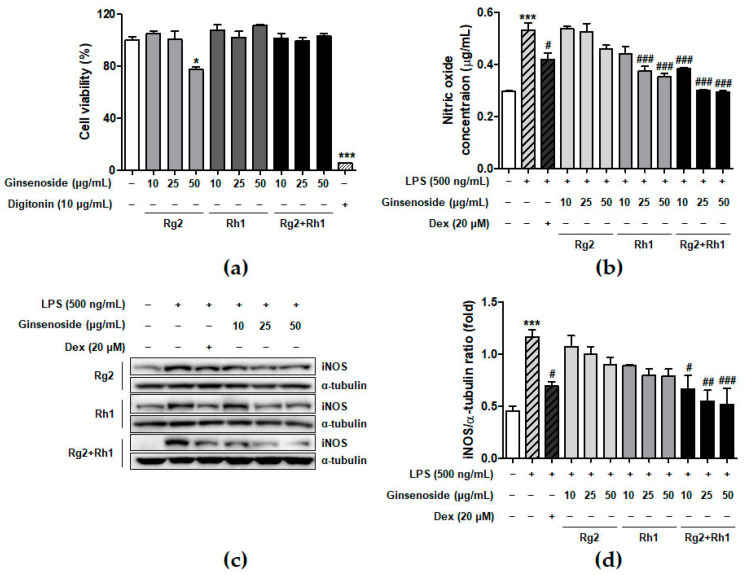
Effect of minor ginsenoside Rg2 and Rh1 on cell viability. (**a**) Cell viability of minor ginsenoside Rg2 and Rh1-treated RAW264.7 cells were measured by 3-(4,5-dimethylthiazol-2-yl)-2,5-diphenyltetrazolium bromide (MTT) assay. The cells were treated with various concentration of Rg2, Rh1, and combination of Rg2 and Rh1 (0 to 50 μg/mL) for 24 h. In the combination of Rg2 and Rh1, Rg2 and Rh1 were mixed with a ratio of 1:1 (10 μg/mL of the combination contains 5 μg/mL Rg2 and 5 μg/mL Rh1, and so forth). Digitonin was used as negative control. (**b**,**c**) The production of nitric oxide was examined by Griess assay (**b**), and expressions of iNOS were evaluated by western blot analysis (**c**). (**d**) The ratio of phosphorylated iNOS/α-tubulin expression represents as the mean ± SEM (*n* = 3). * p < 0.05, *** p < 0.001 compared with control sample, *# p* < 0.05*, ## p* < 0.01, and *### p* < 0.001 compared with lipopolysaccharide (LPS)-treated sample.

To determine the concentration of ginsenosides used in the experiments, we examined the effect of minor ginsenosides on cell viability using 3-(4,5-dimethylthiazol-2-yl)-2,5-diphenyltetrazolium bromide (MTT) analysis (Figure 1a). The RAW264.7 cells were treated with various concentrations of ginsenosides for 24 h. The results showed that Rh1 and the Rg2 and Rh1 combination did not affect cell viability under culture conditions. Next, we determined the effect of minor ginsenosides on NO production (Figure 1b). LPS treatment significantly increased NO production compared to that in the control (no treatment), whereas Rg2, Rh1, or the Rg2 and Rh1 combination treatment decreased LPS-induced NO production in a dose-dependent manner. Of the ginsenosides used in the experiment, the Rg2 and Rh1 combination showed the highest inhibitory effect on NO production (Figure 1b, black bars). Dexamethasone (Dex) was used as a positive control for anti-inhibitory effects of LPS-induced NO production. NO is an inflammatory mediator produced by iNOS [26]. To determine whether the effect of ginsenosides on NO reduction was due to the inhibition of iNOS expression, whole cell lysates were analyzed using western blotting (Figure 1c). LPS treatment markedly increased iNOS protein levels, whereas each ginsenoside inhibited iNOS expression. Specifically, the Rg2 and Rh1 combination displayed the most significant inhibition of iNOS expression in a dose-dependent manner (Figure 1c,d). These data suggest that the ginsenoside Rg2 and Rh1 combination has a superior ability to downregulate LPS-stimulated NO production via the inhibition of iNOS expression. 

### 2.2. Inhibitory Effect of the Ginsenosides Rg2 and Rh1 on LPS-TLR4 Binding on Macrophages

**Figure 2:**
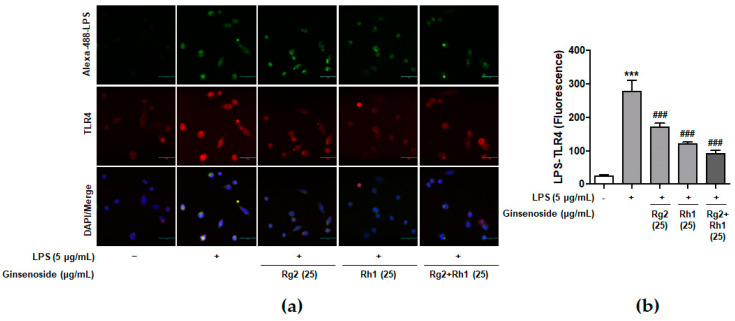
Inhibitory effect of ginsenosides Rg2 and Rh1 on LPS- toll-like receptor 4 (TLR4) binding on macrophages. (**a**,**b**) Peritoneal macrophages were stimulated by Alexa Fluor 488-conjugated LPS for 20 min in the absence or the presence of 25 µg/mL of each ginsenosides or the combination of Rg2 and Rh1. In the combination of Rg2 and Rh1, Rg2 and Rh1 were mixed with a ratio of 1:1 (25 μg/mL of the combination contains 12.5 μg/mL Rg2 and 12.5 μg/mL Rh1). Cells were fixed and incubated with Anti-TLR4 antibody for 3 h followed by incubationwith Alexa Fluor 546 for 1 h. 4′,6-diamidino-2-phenylindole (DAPI) was counterstained for nuclear quantitation. The images were observed using a laser scanning confocal spectral microscope (Nanoscope systems). The bar indicates 30 μm. The relative quantification of the LPS-TLR4 binding was analyzed using Image J software and the expression is represented as the mean ± SEM (*n* = 3). *** p < 0.001 compared with control sample *### p* < 0.001 compared with LPS-treated sample.

Previous reports have suggested that LPS specifically binds to TLR4 on macrophages and induces various intracellular signaling pathways associated with inflammation [7]. To confirm whether ginsenosides inhibited LPS binding to TLR4 on peritoneal macrophages, peritoneal macrophages were treated with Alexa Fluor 488-conjugated LPS in the absence or the presence of each ginsenoside (Figure 2). The LPS treatment activated the cells through the binding of LPS to the TLR4 of peritoneal macrophages, which was repressed by pre-treatment with the ginsenoside Rg2 or Rh1. Interestingly, the ginsenoside Rg2 and Rh1 combination significantly inhibited the binding of LPS to TLR4 in a dose-dependent manner. This result suggests that a combination of the ginsenosides Rg2 and Rh1 has an inhibitory effect on LPS binding to TLR4, which may in turn suppress various cellular signaling transduction pathways induced by LPS-TLR4 binding.

### 2.3. Inhibitory Effect of the Ginsenosides Rg2 and Rh1 on the TLR4-Mediated Signaling Pathways in LPS-Stimulated RAW264.7 Cells 

**Figure 3:**
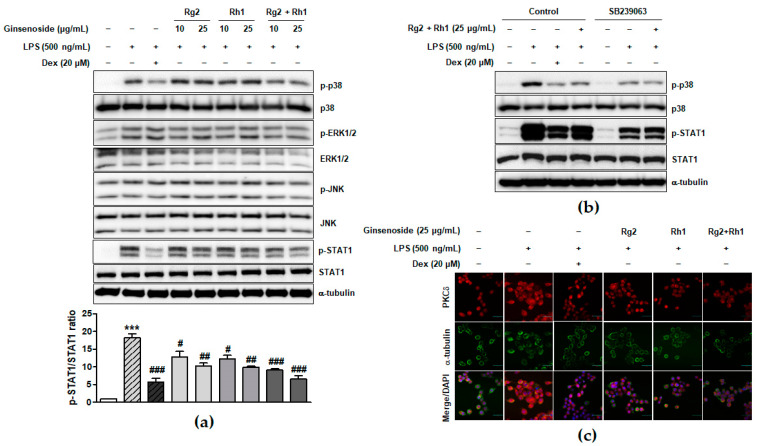
Inhibitory effect of ginsenosides Rg2 and Rh1 on the TLR4-mediated signaling pathways in LPS-stimulated macrophages. (**a**) The cells were pre-treated with ginsenosides Rg2, Rh1, and the combination of Rg2 and Rh1 (10 and 25 μg/mL) or dexamethasone (Dex) for 1 h followed by treatment with LPS (500 ng/mL) for 1 h. In the combination of Rg2 and Rh1, Rg2 and Rh1 were mixed with a ratio of 1:1 (10 μg/mL of the combination contains 5 μg/mL Rg2 and 5 μg/mL Rh1, and so forth). (**a**; lower bar graph) The ratio of phosphorylated STAT1/total STAT1 expression represented as the mean ± SEM (*n* = 3). *** p < 0.001 compared with control sample, *# p < 0.05, ## p* < 0.01, and *### p* < 0.001 compared with LPS-treated sample. (**b**) The cells were pre-treated with SB239063 or 25 μg/mL of ginsenoside Rg2 and Rh1 combination (1:1 ratio) for 1 h followed by treatment with LPS (500 ng/mL) for 1 h. All phosphorylated and total proteins were analyzed by western blotting. (**c**) The RAW264.7 cells were pre-treated with ginsenosides Rg2, Rh1, the combination of Rg2 and Rh1 (1:1 ratio) (25 μg/mL) or dexamethasone (Dex) for 3 h followed by treatment with LPS (100 ng/mL) for 1 h. Immunofluorescence analysis was performed after fixing and immunostaining with PKCδ and α-tubulin. DAPI was co-stained for visualizing nucleus. Images are shown at ×600 magnification. Scale bar indicates 30 μm.

LPS binding-induced TLR4 activation leads to the activation of various intracellular signaling pathways such as MAPK and JAK-STAT signaling pathways, which play an important role in inflammatory responses [8,32]. To investigate the underlying signaling pathways associated with the anti-inflammatory effect of the minor ginsenosides Rg2 and Rh1, MAPK and JAK-STAT1 signaling pathways in LPS-stimulated RAW264.7 cells were examined. As shown in Figure 3a and supplementary Figure S1, the LPS treatment significantly activated ERK1/2, p38, and JNK, whereas treatment with the minor ginsenosides Rg2 and Rh1 significantly inhibited the activation of p38 but not ERK1/2 or JNK in a dose dependent manner. Additionally, treatment with the minor ginsenosides Rg2 and Rh1 completely inhibited LPS-stimulated STAT1 activation in a dose-dependent manner (Figure 3a). Stat1 protein has two isoforms, including Stat1α (91 kDa) and Stat1β (84 kDa) [33]. As shown in Figure 3a, phospho-Stat1 antibody detected the phosphorylated form of p91 Stat1 and the p84 splice variant. To investigate the role of p38 kinase activation on STAT1 serine phosphorylation, LPS-stimulated cells were pre-treated with the p38 specific inhibitor, SB239063 (Figure 3b). LPS-induced STAT1 phosphorylation was reduced by a decrease in p38 phosphorylation by ginsenoside Rg2 and Rh1 (Figure 3b). Since previous reports also suggested the involvement of PKCδ activation in the TLR4-specific signaling pathway, we further investigated PKCδ translocation under LPS and ginsenoside treatment. Figure 3c showed that LPS induced PKCδ translocation to the plasma membrane, but ginsenoside Rg2 and Rh1 treatment reduced LPS-induced PKCδ expression and translocation to the membrane. Especially, the combined ginsenoside treatment further enhanced the blocking effect of LPS-induced PKCδ translocation compared to Rg2 or Rh1 alone treatment. Our data demonstrated that ginsenoside Rg2 and Rh1 are related to the TLR4-specific signaling pathway by downregulating PKCδ translocation and p38-STAT1 activation.

### 2.4. Effect of the Ginsenosides Rg2 and Rh1 on NF-κB p65 Nuclear Translocation and Activation and Cytokine Production

**Figure 4:**
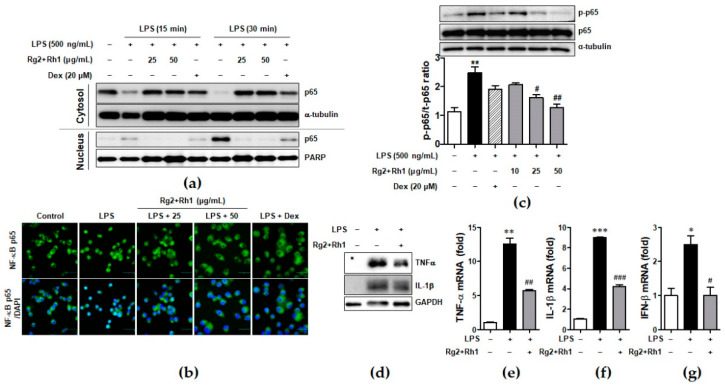
Effect of ginsenosides Rg2 and Rh1 on NF-κB p65 nuclear translocation and activation and cytokine production. (**a**) The RAW264.7 cells were pre-treated with the combination of ginsenosides Rg2 and Rh1 (1:1 ratio) at different concentrations (25 and 50 μg/mL) for 24 h followed by treatment with LPS (100 ng/mL) for 15 or 30 min. Cytosolic and nuclear proteins were extracted and subjected to western blotting. (**b**) The RAW264.7 cells were pre-treated with the combination of ginsenosides Rg2 and Rh1 (1:1 ratio) at different concentrations (25 and 50 μg/mL) for 24 h followed by treatment with LPS (100 ng/mL) for 15 min. Immunofluorescence analysis was performed after fixing and immunostaining with NF-κB p65. DAPI was co-stained for visualizing nucleus. Images are shown at ×600 magnification. Scale bar indicates 30 μm. (**c**) The RAW264.7 cells were pre-treated with the combination of ginsenosides Rg2 and Rh1 (1:1 ratio) at various concentrations (10, 25, and 50 μg/mL) for 1 h followed by treatment with LPS (500 ng/mL) for 3 h. Whole protein lysates were subjected to western blotting. (Lower bar graph) The ratio of phosphorylated p65/α-tubulin expression represents as the mean ± SEM (*n* = 8). (**d**–**g**) Peritoneal macrophages were collected from the mice that were i.p. injected with the combination of ginsenosides Rg2 and Rh1 (1:1 ratio, 20 mg/kg) for 24 h followed by treatment with LPS (10 mg/kg) for 24 h. Inhibitory effects of the ginsenosides on protein expressions of TNF-α and IL-1β were analyzed by western blotting (**d**), and mRNA levels of TNF-α (**e**), IL-1β (**f**), and IFN-β (**g**) were analyzed by qRT-PCR. Each cytokine was normalized by glyceraldehyde 3-phosphate dehydrogenase (GAPDH) (*n* = 3). Data was calculated using the 2^−ΔΔ*C*t^ method and represented as mean ± SEM (*n* = 3). * p < 0.05, ** p < 0.01, and *** p < 0.001 compared with control sample, *# p*< 0.05, *## p* < 0.01, and *### p* < 0.001 compared with LPS-treated sample.

Since NF-κB is essential for regulation of inflammatory responses, we investigated whether the minor ginsenosides Rg2 and Rh1 suppressed NF-κB (p65) activation and nuclear translocation in LPS-stimulated RAW264.7. As shown in Figure 4, they almost inhibited LPS-induced nuclear translocation of NF-κB (p65), as revealed by western blotting (Figure 4a) and immunofluorescence analysis (Figure 4b). Additionally, LPS-stimulated p65 activation was repressed by treatment with the ginsenosides Rg2 and Rh1 (Figure 4c). Next, we further examined the effect of the ginsenosides Rg2 and Rh1 on the induction of pro-inflammatory cytokines such as TNF-α, IL-1β, and IFN-β by LPS in peritoneal macrophages. The peritoneal macrophages, which were collected from the mice injected intraperitoneally (i.p.) with the ginsenosides Rg2 and Rh1 (20 mg/kg) for 24 h, followed by treatment with LPS (10 mg/kg) for 24 h, were subjected to western blotting and qRT-PCR. As shown in Figure 4d, TNF-α and IL-1β protein expression, which was increased by LPS, was reduced by the pre-treatment of the ginsenosides Rg2 and Rh1. Consistently, LPS-induced mRNA levels of TNF-α, IL-1β, and IFN-β were suppressed by the ginsenosides Rg2 and Rh1 (Figure 4e–g).

### 2.5. Effect of the Ginsenosides Rg2 and Rh1 on Pathological Changes and Liver Function in LPS-Challenged Mice

**Figure 5:**
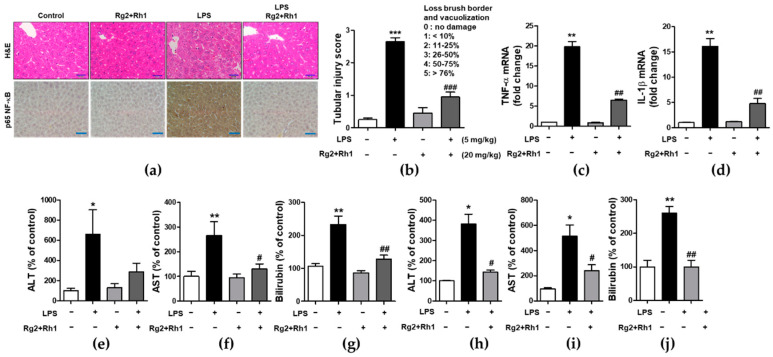
Effect of ginsenosides Rg2 and Rh1 on pathological changes and liver function in LPS-challenged mice. (**a**–**g**) Mice were i.p. injected with ginsenosides Rg2 and Rh1 combination (1:1 ratio, 20 mg/kg) for 2 h followed by treatment with LPS (5 mg/kg) for 6 h. (**a**) Pathological changes of acute liver injury with hematoxylin and eosin (H&E) staining with scale bar 50 μm and immunohistochemical detection of NF-κB p65 expression in mouse liver sections with scale bar 100 μm. (**b**) Bar graph represents histological score for liver sections (*n* = 8). (**c**,**d**) The liver lysates were subjected to qRT-PCR to examine the levels of pro-inflammatory cytokines. (**e**–**g**) The level of serum alanine transaminase (ALT), aspartate transaminase (AST), and bilirubin were detected from mouse serum. (**h**–**j**) Mice were i.p. injected with Rg2 and Rh1 combination (1:1 ratio, 20 mg/kg) for 24 h followed by treatment with LPS (10 mg/kg) for 24 h. The level of serum ALT, AST, and bilirubin were detected from mouse serum. Data are represented as mean ± SEM (*n* = 6). * p < 0.05, ** p < 0.01, and *** p < 0.001 compared with control sample, *# p*< 0.05, *## p* < 0.01, and *### p* < 0.001 compared with LPS-treated sample.

To examine the inhibitory effect of the ginsenosides Rg2 and Rh1 on acute liver damage, mice were i.p. injected with the ginsenosides Rg2 and Rh1 (20 mg/kg) for 2 h or 24 h, followed by treatment with LPS (5 mg/kg) for 6 h or LPS (10 mg/kg) for 24 h. Liver tissues were then harvested and fixed for hematoxylin and eosin (H&E) staining. No histological changes were observed in the control group, whereas the LPS-treated group showed significant histological changes characterized by immune cell infiltration, vacuolation, and necrosis around the central vein area. Notably, the very severe pathological changes induced by LPS were almost entirely prevented by the treatment with the ginsenosides Rg2 and Rh1 (Figure 5a, upper panel, and Figure 5b). Consistent with the inflammatory condition seen in H&E histological data, NF-κB expression level and nuclear localization of NF-κB were much higher in the LPS group than in any other group, whereas, ginsenoside Rg2 and Rh1 treatment completely abolished LPS-induced NF-κB expression in the liver tissue (Figure 5a, lower panel). Since NF-κB is a main transcriptional regulator for pro-inflammatory cytokines, the effects of ginsenoside Rg2 and Rh1 on TNF-α and IL-1β expression in liver tissues were examined. As shown in Figure 5c,d, mRNA levels of LPS-stimulated TNF-α and IL-1β were significantly inhibited by treatment with the ginsenoside Rg2 and Rh1. Likewise, plasma TNF-α and IL-1β levels were significantly increased by LPS stimulation, whereas ginsenoside Rg2 and Rh1 treatment prevented this increase in cytokine levels (Supplementary Figure S2). Moreover, in both the 6 h and 24 h LPS-treated mice, the levels of liver damage markers (aspartate transaminase (AST), alanine transaminase (ALT), and bilirubin) were increased, and treatment with ginsenosides Rg2 and Rh1 significantly reduced these levels (Figure 5e–j). These results indicate that the ginsenosides Rg2 and Rh1 protect liver tissue and function from acute injury by alleviating pathological changes and inhibiting the inflammatory response. 

### 2.6. Effect of the Ginsenosides Rg2 and Rh1 on Pathological Changes and Kidney Function in LPS-Challenged Mice

**Figure 6:**
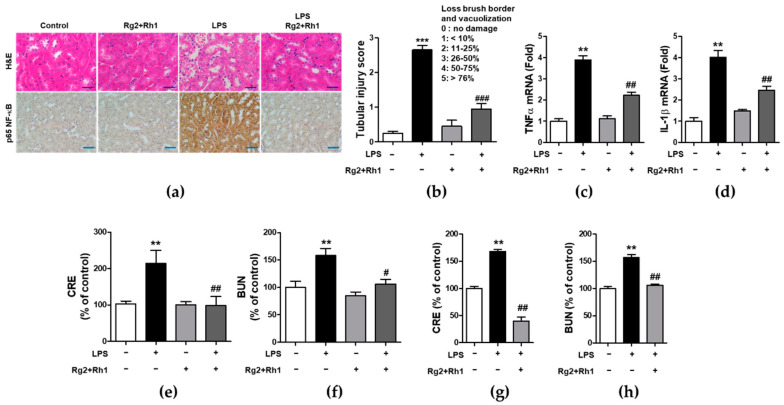
Effect of ginsenosides Rg2 and Rh1 on pathological changes and kidney function in LPS-challenged mice. (**a**–**f**) Mice were i.p. injected with ginsenosides Rg2 and Rh1 combination (1:1 ratio, 20 mg/kg) for 2 h followed by treatment with LPS (5 mg/kg) for 6 h. (**a**) Pathological changes of acute kidney injury with H&E staining with scale bar 50 μm and immunohistochemical detection of NF-κB p65 expression in mouse kidney sections with scale bar 100 μm. (**b**) Histological score for kidney sections (*n* = 8). (**c**,**d**) The kidney lysates were subjected to qRT-PCR to determine the production of pro-inflammtory cytokines. (**e**,**f**) The levels of serum creatinine (CRE) and blood urea nitrogen (BUN) were detected from mouse serum. (**g**,**h**) Mice were i.p. injected with ginsenosides Rg2 and Rh1 combination (1:1 ratio, 20 mg/kg) for 24 h followed by treatment with LPS (10 mg/kg) for 24 h. The levels of serum CRE and BUN were detected from mouse serum. Data are represented as mean ± SEM (*n* = 6). ** *p* < 0.01 and *** *p* < 0.001 compared with control sample, # *p* < 0.05, ## *p* < 0.01, and ### *p* < 0.001 compared with LPS-treated sample.

Next, we evaluated the anti-inflammatory effects of the minor ginsenosides Rg2 and Rh1 on acute kidney injury. Similar to that observed in liver injury, LPS-induced kidney injury was significantly characterized by the following pathological changes: immune cell infiltration, epithelial changes, and necrosis. However, treatment with the ginsenosides Rg2 and Rh1 diminished these changes in kidney tissue (Figure 6a, upper panel, and Figure 6b). Additionally, there was a dramatic increase in NF-κB expression and nuclear localization of NF-κB in the kidney tissues of LPS-treated mice, which was entirely suppressed by ginsenosides Rg2 and Rh1 treatment (Figure 6a, lower panel). Consistently, the increase in the mRNA levels of TNF-α and IL-1β in the kidneys of LPS-treated mice was reduced upon treatment with the ginsenosides Rg2 and Rh1 (Figure 6c,d). Likewise, treatment with the ginsenosides Rg2 and Rh1 also reduced the levels of kidney damage markers (blood urea nitrogen (BUN) and creatinine (CRE)) in the LPS-treated mice for 6 h (Figure 6e,f) and 24 h (Figure 6g,h). Taken together, these results showed that the ginsenosides Rg2 and Rh1 have a protective effect against LPS-stimulated tissue damage in vivo. 

## 3. Discussion

Combination of natural products is a potential strategy that has a variety of potencies and a lower dosage requirement to reduce drug toxicity. This study aimed to investigate the effect of a combination of the minor ginsenosides Rg2 and Rh1 on LPS-stimulated macrophages and acute inflammatory disease in vivo and to demonstrate their synergistic effects on LPS-stimulated inflammation, rather than the efficacy of each.

In this study, Rg2 and Rh1 were combined in a ratio of 1:1, and from a concentration of 10 µg/mL, the combination showed a synergic effect on NO production and iNOS expression, which was greater than the effect of treatment with each compound. As shown in Figure 1b–d, although 10 µg/mL of pure ginsenosides Rg2 or Rh1 did not significantly inhibit NO production and iNOS expression, the combined ginsenosides Rg2 and Rh1 (10 µg/mL) significantly declined both. Especially, the decrease in NO production induced by 10 µg/mL of the combination was relatively equivalent with that induced by ginsenoside Rh1 (25 µg/mL), and the decrease in iNOS expression induced by the combination (10 µg/mL) was more than that induced by ginsenoside Rh1 (25 µg/mL) (Figure 1b–d). Moreover, pure ginsenoside Rh1 has the ability to suppress LPS-TLR4 binding on macrophages. However, the combination of Rh1 and Rg2 exerted more inhibitory effect on the binding between LPS and TLR4 (Figure 2). Furthermore, in Figure 1 and Figure 2, the effect levels of Rh1 alone treatment are shown to be more than those of Rg2 alone treatment. Therefore, a combination of Rg2 and Rh1 with more proportion of Rh1 may lead to better effects, and it is worth investigating in further studies, especially in pharmaceutics field.

A previous study showed that ginsenoside Rg2 protects blood vessels from inflammation by inhibiting the expression of adhesion molecules through inhibition of LPS-stimulated NF-κB [32]. Additionally, the other minor ginsenoside, Rh1, inhibited LPS-stimulated activation of IRAK1 and TAK1 in order to inhibit the phosphorylation of its downstream NF-κB [34]. These results suggest that ginsenosides Rg2 and Rh1 can regulate NF-κB activation via binding to TLR4 in the LPS-induced inflammatory condition. However, there has been no previous study demonstrating the synergistic effects of combined ginsenosides Rg2 and Rh1 in the inflammatory condition. It is also important to identify the binding proteins between LPS and TLR; hence, they can be explored in further studies. 

**Figure 7:**
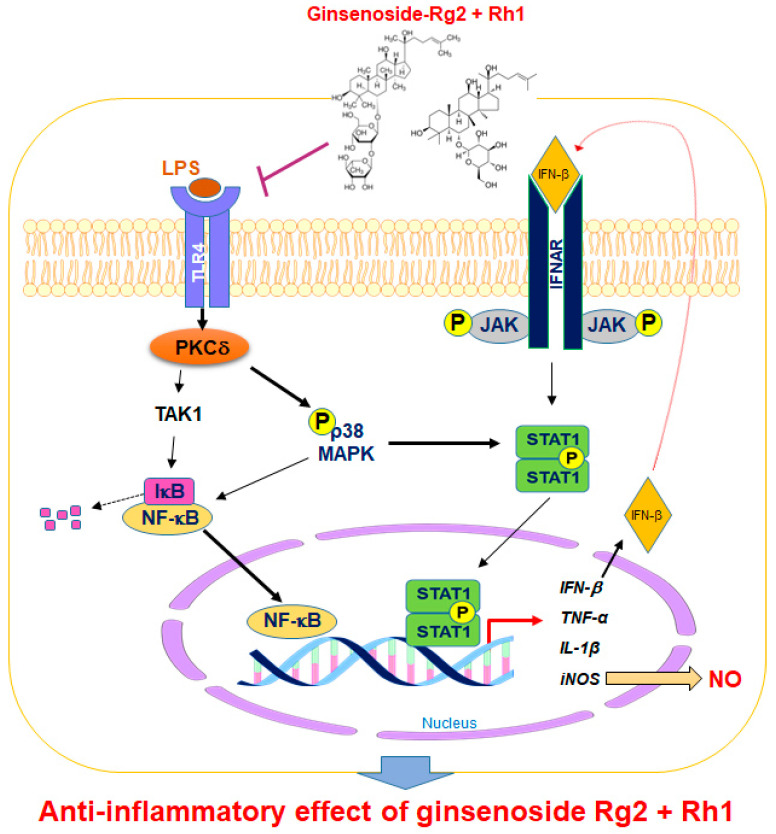
A schematic diagram showing the signaling pathways involved in anti-inflammatory effects of the combination of ginsenosides Rg2 and Rh1 in macrophages. The combination of ginsenosides Rg2 and Rh1 suppressed inflammation by abolishing the binding of LPS to TLR4, thereby inhibiting the TLR4-mediated signaling pathway. The combined ginsenosides Rg2 and Rh1 synergistically blocked LPS-TLR4 signaling-mediated PKCδ translocation to the plasma membrane, resulting in the decrease in p38-STAT1 activation and NF-κB translocation. Additionally, the activations of p38 and IFN-β are associated with the phosphorylation of STAT1, which facilitates the dimer formation of STAT1. This allows STAT1 enter the nucleus and induce iNOS production. The NF-κB translocation to the nucleus caused the transcription of inflammatory cytokines and mediators such as IFN-β, TNF-α, IL-1β, and iNOS, but ginsenosides Rg2 and Rh1 significantly inhibited these inflammatory markers. LPS, lipopolysaccharide; TLR4, toll-like receptor 4; TAK1, transforming growth factor β-activated kinase 1; PKC-β, protein kinase C delta; NF-κB, nuclear factor-kappa B; IκB, inhibitor of kappa B alpha; MAPK, mitogen-activated protein kinase; JAK, janus kinase; STAT1, signal transducer and activator of transcription 1; IFN-β, interferon-beta; iNOS, inducible nitric oxide synthase; IL-1β, interleukin-1 beta TNF-α, tumor necrosis factor-alpha; NO, nitric oxide; P, phosphorylation.

Regulating the JAK-STAT pathway is important in inflammatory diseases. Particularly, STAT1 is stimulated by various cytokines, such as IFN-β and TNF-α, in macrophages [35]. It has been reported that, during an inflammatory response, the activation of p38 MAPK regulates STAT1 phosphorylation at Ser-727 [36]. Additionally, iNOS expression and NO production have been associated with p38 and the STAT1 signaling pathway [37]. In our current study, we demonstrated for the first time that treatment with a combination of the minor ginsenosides Rg2 and Rh1 significantly inhibited LPS-induced phosphorylation of STAT1 and NF-κB p65. Treatment with a combination of the minor ginsenosides Rg2 and Rh1 only inhibited LPS-induced p38 MAPK activation but not that of ERK1/2 and JNK. Interestingly, the combined ginsenosides Rg2 and Rh1 synergistically blocked LPS-TLR4 signaling-mediated PKCδ translocation to the plasma membrane, resulting in p38-STAT1 activation and NF-κB translocation. Additionally, mRNA expression levels of pro-inflammatory cytokines, including TNF-α, IL-1β, and IFN-β, were significantly decreased by the combined ginsenoside treatment. These results suggest that the ginsenosides Rg2 and Rh1 downregulate cytokine production via regulation of NF-κB activation, which may in turn affect the deactivation of LPS-stimulated STAT1 (Figure 7).

In animal inflammatory models, a combination of the ginsenosides Rg2 and Rh1 protected the liver and the kidney from LPS stimulation-induced damages. LPS treatment caused very severe pathological changes in liver and kidneys, whereas the severe pathological changes were inhibited by treatment with the ginsenosides Rg2 and Rh1 as much as the control group. Moreover, there was a reduction in the level of LPS-induced liver and kidney damage indicated by markers such as AST, ALT, bilirubin, CRE, and BUN caused by treatment with the ginsenosides Rg2 and Rh1. In concordance with in vitro data, the increase in the mRNA levels of TNF-α and IL-1β in livers and kidneys of LPS-treated mice was suppressed by the treatment with the ginsenosides Rg2 and Rh1. Activated macrophages are the main producers of inflammatory mediators such as nitric oxide (NO), TNF-α, and IL-1β [38]. Overproduction and prolonged secretion of these mediators and cytokines play a vital role in the development of systemic inflammation, which is associated with tissue and organ damage [39]. Our study demonstrated that ginsenosides Rg2 and Rh1 inhibited the production of inflammatory mediators, including TNF-α, IL-1β, IFN-β, and iNOS, as well as NO production by abolishing the binding of LPS to TLR4. Therefore, ginsenosides Rg2 and Rh1 can inhibit LPS-stimulated inflammation by suppressing macrophage-derived inflammatory mediators to prevent liver and kidney tissue damage in mice.

## 4. Materials and Methods

### 4.1. Materials

Rabbit anti-phospho-ERK1/2 (#9101), rabbit anti-ERK1/2 (#4695), rabbit anti-phospho-p38 (#4631), rabbit anti-p38 (#8690), rabbit anti-phospho-JNK (#9251), rabbit anti-JNK (#9252), rabbit anti-phospho-p65 (#3033), rabbit anti-p65 (#8242), and rabbit anti-phospho-PKCδ (#9374) antibodies were purchased from Cell Signaling Technology, Inc. (Danvers, MA, USA). Rabbit anti-iNOS antibody (#160862) was purchased from Cayman chemical, Inc. (Ann Arbor, MI, USA). Mouse anti-phospho-STAT1 (#sc-136229), mouse anti-STAT1 (sc-417), mouse anti-TNF-α (#sc-52746), hamster anti-IL-1β (#sc-12742), and mouse anti-TLR4 (sc-293072) were purchased from Santa Cruz Biotechnology Inc. (Dallas, TX, USA). Mouse anti-α-tubulin antibody, lipopolysaccharide (LPS, E. coli, O111:B4, #L2630), Griess reagent (#G4410-10G), and ginsenosides Rg2, Rh1, and anti-mouse IgG (#T5393) were purchased from Sigma-Aldrich (St. Louis, MO, USA). Mounting medium with 4′,6-diamidino-2-phenylindole (DAPI) was purchased from Vector Laboratories, Inc. (#Z0215, Burlingame, CA, USA). Phosphate buffered saline (PBS, #EBA-1105) and reverse transcription 5X master mix (#EBT-1511) were purchased from Elpisbio (Daejeon, Korea). Tri-RNA reagent (#FATRR-001) was purchased from Favorgen (Pingtung, China). The 3-(4,5-dimethylthiazole-2-yl)-2, the 5-diphenyltetrazolium bromide (MTT, #M6494), and the Alexa Fluor-488 conjugated LPS (#L23351) were purchased from Invitrogen (Carlsbad, CA, USA). Dulbecco’s modified Eagle’s medium (DMEM, #11963-092) and fetal bovine serum (FBS, #10082147) were purchased from Gibco (Carlsbad, CA, USA). Ginsenosides Rg2 and Rh1 (purity 99%) were purchased from Ace EMzyme (Anseong-si, Kyeonggi-do, Korea). In the combination of Rg2 and Rh1, Rg2 and Rh1 were mixed in a 1:1 ratio, e.g., 10 μg/mL of the combination contained 5 μg/mL Rg2 and 5 μg/mL Rh1, and so forth.

### 4.2. Cell Culture

RAW264.7 cell line murine macrophage was purchased from Korea Cell Line Bank (KCLB, #40071, Seoul, Korea). The cells were maintained by DMEM including 10% fetal-bovine serum (BS), 100 U/mL penicillin, and streptomycin at 37 °C in humidified atmosphere 5% CO_2_ (HERAcell 150i, hermo Electron Corp., Waltham, MA, USA).

### 4.3. Cell Viability Assay

RAW264.7 cell viability was performed using MTT assay. Cells were seeded at the density of 1 × 10^4^ cells/well in 96-well plate. After 24 h of incubation, the cells were starved with fresh FBS free medium for 24 h. The adhered cells were treated with various concentrations (0, 10, 25, and 50 μg/mL) of the compound for 24 h. MTT (12 mM) in FBS-free DMEM media was prepared to treat in each well, and the cells were incubated for 2 h at 37 °C and 5% CO_2_. The medium was removed, and the formazan precipitate was extracted in DMSO. The absorbance was measured at 540 nm on a microplate reader (TECAN, Männedorf, Switzerland).

### 4.4. Confocal Microscopy

Peritoneal macrophages were cultured on a coverglass bottom plate (#30206, SPL, Pocheon-so, Korea) overnight at 37 °C and 5% CO_2_. The cells were pre-treated with each concentration of ginsenosides for 1 h, followed by treatment with Alexa Fluor 488-conjugated LPS (5 μg/mL) for 30 min. The cells were fixed with 4% formaldehyde for 20 min and then stained with rabbit polyclonal anti-TLR4 antibody (Santa Cruz Biotechnology) for 3 h at 4 °C and incubated with secondary antibodies conjugated with Alexa Fluor-546 for 1h. The stained cells were examined with a laser scanning confocal spectral microscope (K1-Fluo, Nanoscope systems, Daejeon, Korea).

### 4.5. NO Measurement

The amount of NO in the culture media was determined by Griess reagent. RAW264.7 cells were seeded in the 96-well plate (2 × 10^4^ cells/well) and incubated at 37 °C with 5% CO_2_ for 24 h. After cells were starved for 4 h, the cells were pre-treated with Rg2 and Rh1 for 1 h followed by stimulation by 500 ng/mL LPS for 18 h. Then, incubated media were transferred into a new 96-well plate, and equal volumes of Griess reagent were mixed and incubated for 10 min at dark condition. The absorbance was measured at a wavelength of 540 nm using the micro plate reader (TECAN, Männedorf, Switzerland).

### 4.6. Western Blotting

RAW264.7 cells were pre-treated with vehicle or 10, 25, or 50 μg/mL Rg2 and Rh1 followed by treatment with 500 ng/mL of LPS. After treatment, cells were washed with PBS, and lysates were prepared using 2X SDS lysis buffer including 1 M Tris-HCl (pH 7.4), 25% Glycerol, 10% SDS, 5% 2-mercaptoethnol, and 1% bromphenol blue. The protein extracts were resolved by sodium dodecyl sulfate polyacrylamide gel electrophoresis (SDS-PAGE), electro-transferred onto a Hybond enhanced chemiluminescence nitrocellulose membrane, and visualized by using the enhanced chemiluminescence detection reagents (Amersham Pharmacia Biotech, Piscataway, NJ, USA) according to the manufacturer’s instructions. Each protein level was detected by western blotting with each corresponding specific antibody.

### 4.7. Immunofluorescence Staining

RAW264.7 cells were cultured on a coverglass bottom plate (#30206, SPL, Pocheon-so, Korea) overnight at 37 °C and 5% CO2. After the treatment, the cells were fixed with 4% formaldehyde for 10 min. For permeabilization, cells were incubated with 0.2% Triton X-100 for 10 min at room temperature and then washed three times in PBS. Cells were blocked with 3% bovine albumin serum for 30 min at room temperature, incubated with primary antibody as indicated overnight at 4 °C, and then incubated with fluorescent secondary antibody for 1 h at room temperature. The stained cells were observed under a laser scanning confocal spectral microscope (K1-Fluo, Nanoscope systems, Daejeon, Korea).

### 4.8. Preparation of Cytosolic Extracts and Nuclear Extracts

The RAW264.7 cells were pre-treated with ginsenosides Rg2, Rh1, or Rg2 and Rh1 combination (Rg2 and Rh1 were mixed in a 1:1 ratio) (25 and 50 μg/mL) for 24 h followed by treatment with LPS (100 ng/mL) for 15 or 30 min. Cytosolic and nuclear proteins were prepared as previously described [40]. The extracts were then subjected to western blotting.

### 4.9. Real-Time Reverse Transcription (RT)-PCR Reaction

The RAW264.7 cells were seeded in a 6-well plate at the density of 1 × 10^6^ cells/well and incubated at 37 °C with 5% CO_2_ for 24 h. The cells were pre-treated with Rg2 and Rh1 for 1 h followed by treatment with 500 ng/mL LPS for 6 h. The qRT-PCR assay was used to analyze the mRNA expression of inflammatory cytokine genes, such as TNF-α and IL-1β, according to the previous report [41]. Total RNA was isolated from cells using Tri-RNA reagent according to the manufacturer’s instructions. One μg of total RNA in each group was reverse transcribed into single-stranded cDNA using the reverse transcription 5X master mix. The mRNA expression of the genes was quantified with iQ™ SYBR green supermix (#170-8882, Bio-rad Inc., Hércules, CA, USA) using a CFX Connect™ (Bio-rad Inc.). The relative gene expressions were calculated using the 2^−ΔΔ*C*t^ method, and all primer sequences used in qRT-PCR experiments are listed as follows: TNF-α; Forward-5′-GCCTCTTCTCATTCCTGCTTG-3′, Reverse-5′-CTGATGAGAGGGAGGCCATT-3′, IL-1β; Forward-5′-AACCTGCTGGTGTGTGACGTTC-3′, Reverse-5′- CAGCACGAGGCTTTTTTGTTGT-3′, GAPDH; Forward-5′-AACGTCATCCCAGAGCTGAA-3′, Reverse-5′-CTGCTTCACCACCTTCTTGA-3′, IFN- β; Forward-5′-TCACCTACAGGGCGGACTTC-3′, Reverse-5′-TCTCTGCTCGGACCACCATC-3′.

### 4.10. Animal Experiments

ICR mice (male) were purchased from SAMTAKO Inc. (Osan-si, Kyeonggi-do, Korea). The mice were acclimated for a week before the experiments and fed with standard mice feed under the 23 ± 2 °C, 12:12 dark to light conditions. All animal studies were performed with the approval from Institutional Animal Care and Use committee of Chungnam National University (CNU-00797). Mice were randomly divided into four groups (group 1: vehicle group; group 2: 5 mg/kg LPS; group 3: 20 mg/kg Rg2 and Rh1 combination (Rg2 and Rh1 were mixed in a 1:1 ratio); group 4: 20 mg/kg Rg2 and Rh1 combination + 5 mg/kg LPS, *n* = 8 in each group). Mice were administered complex Rg2 and Rh1 at a dosage of 20 mg/kg diluted in distilled water containing 0.01% DMSO for 2 h by intra-peritoneal injection except groups 1 and 2. After 2 h, the mice of group 2 and 4 were damaged by intra-peritoneal injection of 5 mg/kg LPS diluted in PBS for 6 h. Then, mice were sacrificed, and tissues and blood were collected. Serums were isolated from blood by centrifugation at 4000 rpm at room temperature (RT) and stored at −80 °C for bio-chemistry analysis. Tissues were fixed in 4% formalin solution and embedded in paraffin for histological analysis.

### 4.11. Isolation of Peritoneal Macrophages

Peritoneal macrophages were collected from the mice by infusing their peritoneal cavity with 5 mL ice-cold DMEM including 10% fetal-bovine serum (FBS), 100 U/mL penicillin, and streptomycin. Collected cells were centrifuged, and the cell pellets were resuspended in the complete media. Cell number was determined under microscopy using trypan blue exclusion. According to each experiment, the number of cells was adjusted and then incubated at 37 °C under 5% CO_2_ in a humidified incubator. 

### 4.12. Histological Analysis

Dissected liver and kidney tissues were fixed in 4% formalin solution followed by dehydration by passing through a series of graded alcohol and were finally embedded in paraffin. Then, 3 μm thick sections of the tissue were cut using automated rotary microtome and were stained with H&E stain. Digital images were captured using the light microscope (Olympus IX71, Tokyo, Japan). To evaluate the degree of pathological changes on acute tissue injury, levels of immune cell filtration, tissue damage, and necrosis were scored by random investigators. The degree of tissue injury was scored as follow: grades 0, 1, 2, 3, and 4 represent no damage, mild change, moderate change, severe change, and very severe change, respectively. All scores were determined by three independent observers in a blinded manner.

### 4.13. Immunohistochemistry Staining

The 3 μm thick sections of the tissue were used. After deparaffinization and rehydration, the tissue sections were incubated with 1% hydrogen peroxide for 10 min. Then, 5% bovine serum albumin solution was applied to prevent nonspecific staining. Then, the sections were incubated with primary antibody at room temperature for 2 h. The biotinylated secondary antibody, the avidin-biotin complex (ABC), and the 3,3′-Diaminobenzidine (DAB) chromogens were then applied. Sections were counterstained with hematoxylin. After dehydration, sections were analyzed using the light microscope (Olympus IX71, Tokyo, Japan).

### 4.14. Biochemical Analysis

Blood samples were obtained from LPS-injected mice before harvest. Blood samples were collected (0.5 mL) and put into vacutainer tubes without anticoagulant for serum separation. The tubes were maintained at room temperature for at least 90 min. Then, serum of mice was separated using centrifuge at 4000 rpm for 10 min. To analyze the level of total bilirubin, AST, ALT, CRE, and BUN, each serum was analyzed by clinical blood analyzer (FUJI DRI-CHEM 7000i, Tokyo, Japan) using each parameter kit (FUJIFILM, Tokyo, Japan).

### 4.15. Statistical Analysis

All data are presented using GraphPad Prism 5 (version 5.02, GraphPad Software Inc., San Diego, CA, USA) for statistical analysis. One-way analysis of variance (ANOVA) followed by a Bonferroni multiple comparison was performed. A *p* value < 0.05 was considered significant. All experiments were expressed as the mean ± SEM and were performed independently at least 3 times.

## 5. Conclusions

Our study demonstrated that the minor ginsenosides Rg2 and Rh1 exerted anti-inflammatory effects on LPS-stimulated acute inflammation. They regulated TLR4-mediated intracellular signaling pathways such as PKCδ translocation, JAK-STAT1, and MAPK, especially through the activation of STAT1-p38 and NF-κB. Additionally, they inhibited the production of pro-inflammatory cytokines in LPS-stimulated macrophages. Notably, they protected tissue functions from acute liver and kidney injuries in mice. Therefore, our results suggest that a combination of the ginsenosides Rg2 and Rh1 has superior anti-inflammatory effects compared to each ginsenoside alone. This information may be used to create more potent herbal medicines against systemic bacterial infections.

## Supplementary Materials

The following are available online at https://www.mdpi.com/1422-0067/21/18/6656/s1, Figure S1: Effect of ginsenosides Rg2 and Rh1 on activation of MAPK signaling in LPS-stimulated macrophages; Figure S2: Inhibitory effect of Rg2 and Rh1 on cytokine productions in plasma of LPS-stimulated mice.

## Abbreviations

ALT	Alanine transaminase
AST	Aspartate transaminase
BUN	Blood urea nitrogen
CRE	Creatinine
ERK 1/2	Extracellular signal-regulated kinase 1/2
FBS	Fetal bovine serum
H&E	Hematoxylin and eosin
IL-1β	Interleukin-1 beta
iNOS	Inducible nitric oxide synthase
JAK	Janus kinase
JNK	c-Jun N-terminal kinase
LPS	Lipopolysaccharide
MTT	3-(4,5-dimethelthiazon-2-yl)-2,5-diphehnyltetrazolium bromide
NF-κB	Nuclear factor kappa-light-chain-enhancer of activated B cells
NO	Nitric oxide
PBS	Phosphate buffered saline
PPT	Protopanaxatriol
PPD	Protopanaxadiol
STAT1	Signal transducers and activator of transcription
TLR4	Toll-like receptor 4
TNF-α	Tumor necrosis factor-alpha
