# Supplementary material for: Minor Ginsenoside Rg2 and Rh1 Attenuates LPS-Induced Acute Liver and Kidney Damages via Downregulating Activation of TLR4-STAT1 and Inflammatory Cytokine Production in Macrophages
Source: Int J Mol Sci. 2020 Sep 11;21(18):6656. doi: 10.3390/ijms21186656 (PMC7555743; doi:10.3390/ijms21186656)
Supplement: Supplementary file 1 [file ijms-21-06656-s001.pdf]

Supplementary material

Enzyme-linked immunosorbent assay (ELISA)

Levels of TNF-α and IL-1β in plasma was measured using an ELISA kits (R&D, Minneapolis, MN) according to the manufacturer's instructions. Briefly, 50 μl of biotinylated antibody reagent and the samples were added to the anti-mouse TNF-α and IL-1β precoated 96-well strip plates. The plates were covered and kept at room temperature for 2 h and washed three times followed by the addition of 100 μl of Streptavidin–HRP concentrate. After adding 100 μl of TMB substrate solution, the reaction was quenched by adding 100 μl of TMB stop solution, and the absorbance of the plates was measured at 450 nm to 550 nm using a microplate reader. A standard curve was run on each assay plate using recombinant TNF-α and IL-1β in serial dilutions.

Supplementary figure legend

Figure S1. Effect of ginsenosides Rg2 and Rh1 on activation of MAPK signaling in LPS-stimulated macrophages. Cells were pre-treated with ginsenosides Rg2, Rh1, and the combination of Rg2 and Rh1 (10, and 25 μg/mL) or dexamethasone (Dex) for 1 h followed by treatment with LPS (500 ng/mL) for 1 h. In the combination of Rg2 and Rh1, Rg2 and Rh1 were mixed with a ratio of 1:1 (10 μg/mL of the combination contains 5 μg/mL Rg2 and 5 μg/mL Rh1, and so forth). The ratio of phosphorylated STAT1/total STAT1 expression represents as the mean ± SEM ($n = 3$). ***$p < 0.001$ compared with control sample, #$p < 0.05$, ##$p < 0.01$, and ###$p < 0.001$ compared with LPS-treated sample.

Figure S2. Inhibitory effect of Rg2 and Rh1 on cytokine productions in plasma of LPS-

stimulated mice. Data are represented as mean ± SEM ($n = 6$). **$p < 0.01$ compared with control sample, ##$p < 0.01$ compared with LPS-treated sample.

Supplementary figures

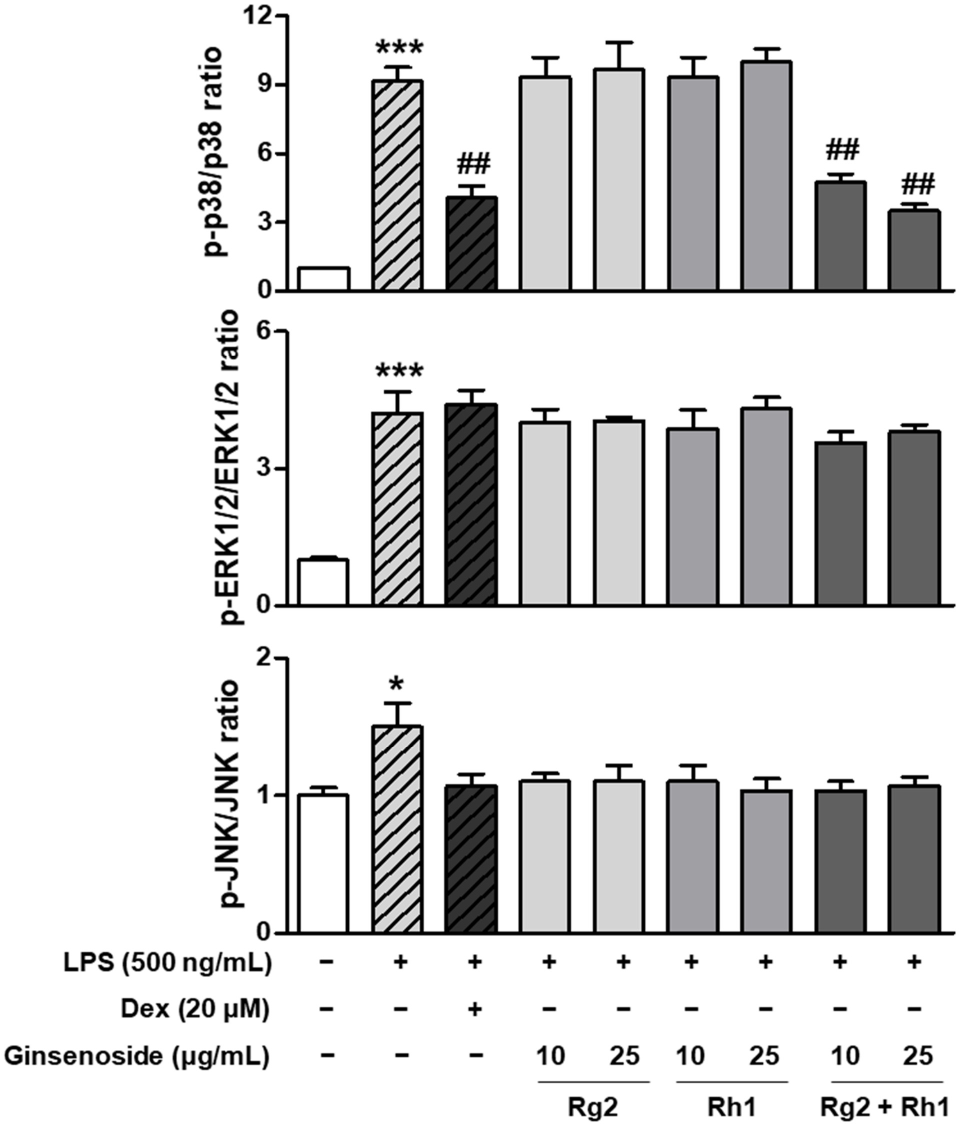

Figure S1

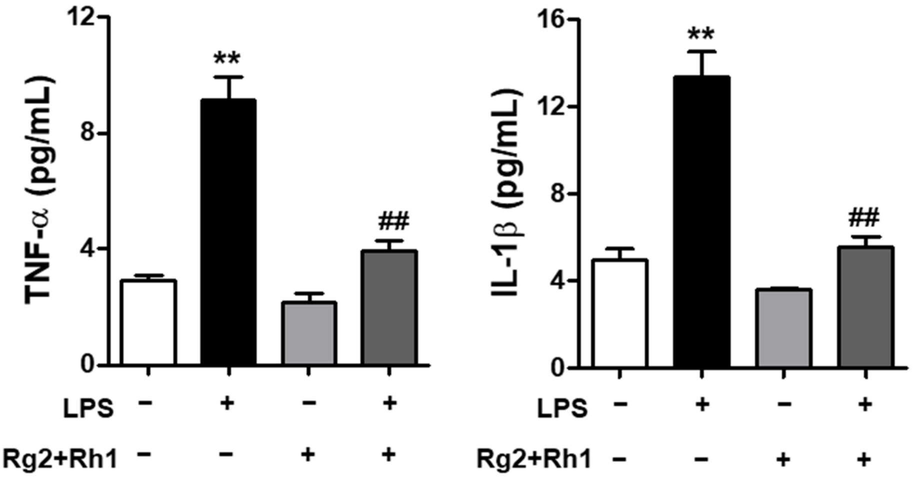

Figure S2
